# Strengthening evidence-based practices in assessment and treatment planning for substance use disorder: an evaluation of a Swedish training program for social services

**DOI:** 10.3389/fpsyt.2026.1834745

**Published:** 2026-06-17

**Authors:** Lena M. Lundgren, Wossenseged Birhane Jemberie, Mikael Sandlund, Siv Nyström, Marcus Blom Nilsson, Robert Grahn

**Affiliations:** 1 Department of Social Work, Umeå University, Umeå, Sweden; 2 Cross-National Behavioral Health Laboratory, Graduate School of Social Work, University of Denver, Denver, CO, United States; 3 Centre for Demography and Aging Research (CEDAR), Umeå University, Umeå, Sweden; 4 Psychiatry Unit, Department of Clinical Science, Umeå University, Umeå, Sweden; 5 Independent Researcher, Stockholm, Sweden

**Keywords:** Addiction assessment, continued professional education, evidence-based addiction treatment assessment implementation, evidence-based practice, immersion training, improving practitioners’ capacity, improving use of technology in SUD assessment

## Abstract

**Background and aim:**

Social workers regularly encounter clients affected by substance use disorders (SUD), yet training in evidence-based assessment and interventions remains limited. In Sweden, municipal social workers have statutory responsibility for SUD assessment, intervention planning and client follow-up which underscores the need for targeted professional education. This study aimed to: (i) describe social workers’ baseline self-assessed competence in evidence-based practices for SUD and its associations with demographic and professional characteristics, and; (ii) examine knowledge gains associated with completion of an evidence-based educational intervention.

**Methods:**

A non-randomized pre–post design included 173 municipal social workers recruited from 64 municipalities across five cohorts (2021–2023). Baseline self-assessed competence was analyzed using ordinal logistic regression, and knowledge gains were assessed using module-specific pre–post items with paired *t*-tests with Cohen’s *d*.

**Results:**

Baseline competence was rated as low to moderate, with professional experience predicting higher perceived competence. Substantial baseline knowledge gaps were observed, particularly in evidence-based psychosocial interventions and use of digital technology in assessment and planning of treatment. Knowledge increased significantly across all modules after training completion (*d* = 0.26–1.41). The largest knowledge gains were for modules on use of technology in the assessment and treatment-planning, biopsychosocial interventions, research-informed use of the assessment tool Addiction Severity Index (ASI), and the professional role in assessment and care planning. Score variability decreased across several modules.

**Conclusion:**

Completion of an immersive, online, evidence-based training was associated with meaningful improvements in social workers’ knowledge related to substance use assessment, intervention planning, and use of technology. Although causal inference is limited by the non-randomized single-group pre–post design, the findings support the potential of scalable educational interventions to address competence gaps in substance use services even in organizations with high caseloads, workforce turnover, and limited training opportunities.

## 1 Introduction

### 1.1 Background

Social workers across a wide range of practice settings regularly encounter clients affected by substance use disorders (SUDs). This includes not only specialized addiction services but also core areas of municipal social work such as housing and supported living services, social assistance, child and family services, mental health care, home care and care home services. As a result, social workers are frequently required to assess substance use–related risks, needs, and service eligibility, even when SUDs are not the primary reason for contact. Moreover, because substance use problems often co-occur with mental health conditions, social vulnerability, chronic health needs, and involvement with multiple care services, effective practice requires competencies that support interdisciplinary and collaborative care provision across services. Population-based epidemiological evidence indicates that individuals with substance use disorder are approximately two to four times more likely than the general population to also meet criteria for mood or anxiety disorder, with the strongest associations observed for illicit drug dependence and major depression, and consistently larger effects for dependence than for abuse (1). Comorbidity rates are typically even higher in clinical samples, with up to 50% of individuals with substance use disorders also living with a mental health condition (2). Together, these complex demands underscore the need for foundational competencies in substance use assessment, follow-up, and planning for evidence-based and interdisciplinary treatment across areas of behavioral health (substance use disorders and mental health disorders), physical health, and social determinants of health such as family, employment, education, and housing (3, 4).

Yet, persistent knowledge gaps in substance use assessment, evidence-based intervention, and follow-up have been documented among social workers both in Sweden and internationally (5–9). One contributing factor is the historically limited emphasis on substance use disorders and evidence-based treatment approaches within general social work education, leaving many practitioners insufficiently prepared to identify, assess, and address substance use problems in routine practice (7, 10–12).International research indicates that structured and targeted training can improve knowledge, confidence, and preparedness for substance use assessment and intervention. Faculty-focused training programs have demonstrated promise, with brief, intensive in-person training associated with strengthened competence in screening, brief intervention, medication-based treatment, and relapse prevention within social work education (13). Subsequent adaptations of these models suggest that online “train-the-trainer” formats can provide a scalable and cost-effective approach, yielding significant improvements in substance use–related teaching capacity and preparedness among multidisciplinary health professions educators, including social work, nursing, medical faculty (14).

Similarly, the integration of Screening, Brief Intervention, and Referral to Treatment (SBIRT), an evidence-based approach for the prevention and early detection of risky substance use, within core social work curricula has been associated with sustained improvements in students’ substance use–related knowledge, confidence, and professional attitudes toward working with individuals with risky substance use (15–18). More recent curricular interventions within Master of Social Work programs likewise point to gains in substance use–related knowledge and client-centered practice skills (11), while emerging evidence from paraprofessional training initiatives suggests that targeted and interdisciplinary instruction can strengthen both knowledge and self-efficacy in evidence-based approaches, including motivational interviewing and harm reduction (19).

### 1.2 The Swedish social services responsibility for assessment-treatment planning for SUD

In Sweden, responsibility for assessment of risky substance use and for the provision of, or referral to, addiction treatment has traditionally rested with municipal social services, which are embedded within local public administrations and governed by elected municipal assemblies. Social workers employed by the municipalities are responsible for assessing needs, making service decisions, planning interventions, and conducting follow-up in accordance with the national Social Services Act. Addiction treatment may be voluntary or, where legally mandated, provided under compulsory care following formal needs assessments and judicial decisions. Clients may self-refer or be referred to municipal social services for risky substance use or substance use disorders. Assessment, treatment, and follow-up services are publicly funded and available to all registered residents through municipal social services.

Municipal addiction services in Sweden are characterized by substantial organizational variation (20). Municipalities differ in whether services are provided in-house or purchased from non-public providers, in the degree of specialization versus generalist practice, and in how addiction services are organized in relation to other welfare domains such as mental health, disability services, social psychiatry, or housing support. Smaller municipalities more often rely on generalist models, while larger municipalities tend to offer specialized outpatient units and a broader range of evidence-based psychosocial interventions.

As of July 2025, a revised Social Services Act has entered into force, reinforcing and clarifying the statutory responsibility of social services to actively offer support and care to individuals with harmful use or dependence, including alcohol, drugs, medications, doping substances, and gambling. The revised law places increased emphasis on early intervention, preventive work, careful follow-up of planned interventions, and the delivery of social services based on scientific knowledge and proven experience. While implementation will take time, the updated legal framework further strengthens the formal responsibility of social services and social workers in substance use prevention, assessment, intervention planning, and follow-up.

Despite this central and increasingly explicit responsibility, there is limited systematic knowledge about the composition and competence of the addiction treatment workforce in Sweden. Available data from the National Board of Health and Welfare point to substantial diversity in the educational backgrounds of staff in municipal outpatient addiction services, including social workers, treatment assistants, nurses, and other personnel with varying levels of formal training. Across service settings, competence requirements differ substantially, and in residential and housing-based services, formal educational standards beyond managerial roles are limited.

Since the early 1990s, the National Board of Health and Welfare has recommended the Addiction Severity Index (ASI) as the primary assessment tool for adults with substance use problems, due to its biopsychosocial scope, reliability, validity and applicability across diverse population and service contexts. The ASI has been integrated into a national digital platform that enables real-time access to assessment data, longitudinal follow-up, and aggregation of client information across municipalities. In principle, this infrastructure provides strong support for standardized assessment, documentation, and evidence-informed service planning, aligning well with both earlier and current legal requirements regarding follow-up and quality assurance. However, research based on national ASI data has shown that, despite high-quality intake assessments, follow-up interviews are infrequently conducted, and assessment data are not consistently used to inform clinical decision-making (7). These findings suggest that the existence of standardized tools and digital infrastructure alone is insufficient to ensure their systematic and clinically meaningful use in routine practice. While educational efforts alone cannot eliminate all organizational barriers, targeted training is a necessary component for strengthening professionals’ capacity to translate assessment data into follow-up, planning, and intervention.

A further and longstanding challenge concerns individuals with co-occurring substance use disorders and psychiatric conditions. This group has repeatedly been identified as experiencing fragmented care, with responsibility divided between municipal social services and regional health care, often resulting in delayed or discontinuous interventions. National inquiries have highlighted these coordination problems and proposed reforms aimed at clarifying responsibility and strengthening integrated care. In 2025, a dedicated national delegation was appointed to further develop an implementation strategy, reflecting both the urgency and complexity of reform in this area. During this ongoing transition, municipal social services continue to play a central role in assessment, coordination, prevention, and service planning for individuals with complex and co-occurring needs.

### 1.3 Aim of the study

Against the backdrop of persistent knowledge gaps, organizational variation, evolving legal requirements, and increasing expectations for evidence-based and coordinated substance use services within Swedish municipal social services, the present study had two aims: **(i)** to describe baseline levels of self-assessed competence in substance use practice among municipal social workers, specifically related to work with different client groups and the use of evidence-based practices, and to examine associations with demographic and professional characteristics, and; **(ii)** to evaluate whether participation in an intensive, online, evidence-based immersion training program was associated with improvements in knowledge related to substance use disorders, client assessment, evidence-based intervention planning, follow-up, and the use of digital decision-support systems.

## 2 Materials and methods

### 2.1 Study design

This study used a non-randomized, single-group pre–post design to evaluate changes in social workers’ knowledge related to SUDs and evidence-based practices following participation in an educational intervention. In addition, baseline cross-sectional analyses were conducted to examine associations of between training participants’ demographic and professional characteristics and their self-assessed competences both with respect to working with specific client groups and evidence-based practices. This study was approved by the Swedish Ethical Review Authority (DNR: 2019-06459). All participants provided informed consent prior to participation. All data were deidentified before analysis to ensure participant confidentiality.

### 2.2 Participants and recruitment strategy

Participants were municipal social workers employed in Swedish social services with professional responsibility for assessing and planning services for adults with risky substance use or substance use disorders. Participants were eligible for inclusion if they:

1. had, or were expected to obtain, access to a web-based assessment and management information system named Net-Klient;
2. were professionally responsible for assessing adults with risky or severe substance use;
3. had received formal managerial approval to participate in both the training and the associated research study; and
4. provided written consent after being informed that participation in the educational program was independent of participation in the research project.

Recruitment was conducted in collaboration with regional research and development (R&D) units in social work across Sweden. Recruitment information was disseminated through R&D networks, the Swedish National Board of Health and Welfare, Umeå University (the educational provider), and the Net-Klient care management information system. Initially, recruitment focused on municipalities in northern Sweden (Norrbotten, Västerbotten, Västernorrland, Jämtland, and Gävleborg), where geographic distance has historically limited access to continuing professional education. Due to COVID-19 restrictions, the course was delivered fully online. After the first year, recruitment was expanded nationally with social workers from 64 of Sweden’s 290 municipalities enrolling in the program.

The initial pre-pandemic recruitment target was 240 participants. Pandemic-related delays in recruitment and the transition to fully online delivery required a reduction of the target intake to 215 participants. The first 42 participants, admitted in January 2021, took part in a feasibility and usability study which was used to refine course content and assess acceptability and fidelity and were therefore excluded from the present analyses. The final analytic sample consisted of 173 participants admitted across five cohorts between August 2021 and January 2023, all of whom completed baseline and follow-up assessments and received identical training.

### 2.3 Research program and research team description

The educational intervention was developed within the six-year interdisciplinary research program *Education and Systematic Service Assessment through Technology and Research to improve the effectiveness of the Swedish Social Welfare System (ESTR)*, funded by the Swedish Research Council on Health, Welfare and Work (Forte). ESTR is structured in sequential phases. The first phase examines whether participation in the educational intervention is associated with gains in social workers’ knowledge and confidence related to substance use disorders, including client assessment and follow-up using the Addiction Severity Index (ASI), evidence-based intervention planning, and the use of digital decision-support systems. Subsequent phases examine whether course participation is associated with increased use of assessments, follow-up interviews, and other evidence-based methods at the social worker and municipal levels, and whether increased implementation of these practices is associated with changes in client-level outcomes using large-scale registry data. The present article addresses the first phase of the ESTR program, focusing on whether participation in the educational intervention was associated with improvements in knowledge related to substance use assessment, evidence-based intervention planning, and follow-up practices.

The ESTR research team was cross-disciplinary and included social work researchers and educators with prior professional experience in municipal social services and addiction treatment, a clinical psychiatrist with expertise in substance use disorders who developed the medical treatment modules, a public health specialist with experience in complex interventions, a certified ASI trainer, and computer engineers with experience in developing management information systems for social services. The program was further supported by an advisory group consisting of practicing social workers, individuals with lived experience of municipal social services, regional R&D representatives, an expert from the Swedish National Board of Health and Welfare (NBHW), and professionals with experience implementing new or improved practices in municipal settings.

### 2.4 Description of the educational intervention: the effective planning of interventions course

The Effective Planning of Interventions (EPI) course was developed to strengthen social workers’ competencies in substance use assessment, evidence-based intervention planning, interdisciplinary collaboration, and the use of digital decision-support systems. Implemented between 2021 and 2023, the course built on international educational models such as an NIAAA-funded immersion training for social work faculty (13), while being adapted to the organizational, legal, and cultural context of Swedish municipal social services.

The EPI course development involved collaboration between social work practitioners, researchers, and educators, addiction psychiatry experts, and digital health technology specialists. Representatives from the NBHW participated in curriculum development to ensure alignment with national ASI guidelines and Swedish higher education standards, while maintaining relevance for frontline practice.

The curriculum integrated several complementary theoretical frameworks. A biopsychosocial perspective provided a foundation for understanding the causes and consequences of substance use disorders. Evidence-based practice principles guided the selection of assessment tools and interventions, emphasizing the integration of empirical evidence, professional judgment, and client preferences. In addition, digital practice frameworks from health and social care informatics informed the course’s approach to using structured data systems for assessment, outcome monitoring, and care planning.

The EPI course comprised ten modules delivered over three and half intensive online training days, followed by an additional one and a half days of structured reflection, examination, and implementation follow-up. Eight modules focused on knowledge acquisition and were assessed using a pre–post design, while two applied modules addressing outcome monitoring and integrated care planning emphasized experiential learning and reflective activities. Follow-up sessions were conducted after participants returned to practice to discuss implementation experiences and identify barriers and facilitators to applying new knowledge. **Table 1** provides an overview of each module, detailing contributors, core content, and learning objectives.

**Table 1:** Overview of training modules.

Module	Contributors	Core content	Learning objectives
*Professional Role in Assessment and Care Planning*	Professor (Evidence-based social work)	Social work practices, history of addiction care, biopsychosocial approaches, continuity of care, ASI framework.	1. Understand the role of social workers in ASI. 2. Apply biopsychosocial perspectives in assessment.
*Alcohol and Drug Use: Causes and Consequences*	Professor (Clinical psychiatry)	Physiological mechanisms, biopsychosocial impacts, and societal consequences of substance use.	1. Identify and explain mechanisms of SUD. 2. Analyze individual and societal impacts of substance use
*Evidence-Based Biopsychosocial Interventions: Part I*	Professor (Evidence-based social work)	Empirical support for psychosocial and pharmacological interventions.	1. Evaluate evidence supporting psychosocial and pharmacological interventions. 2. Compare different evidence-based intervention approaches.
*Evidence-Based Biopsychosocial Interventions: Part II*	Professors (Evidence-based social work; Clinical psychiatry)	Pharmacological treatments and interdisciplinary evidence-based interventions.	1. Explain principles of medication-based treatment for SUD. 2. Evaluate the evidence supporting MBT in clinical practice.
*ASI from a Research Perspective*	Professor (Evidence-based social work); empirical researchers (ASI data analysis)	Swedish ASI data findings, predictive reliability, population-specific outcomes.	1. Analyze Swedish ASI research findings. 2. Evaluate predictive reliability of ASI.
*ASI for Follow-Up Assessment, feedback and clinical decision support*	Trainer (ASI implementation specialist)	Importance of follow-up assessments, engagement strategies, monitoring progress.	1. Apply ASI in follow-up assessments and feedback processes. 2. Use follow-up data to support clinical decision-making and client engagement.
*Integrating MI and Relapse Prevention in Care Planning*	Lecturer in Social Work (Therapeutic methods specialist)	Integration of MI and relapse prevention into follow-up and treatment planning.	1. Apply MI techniques in assessments and follow-up contexts. 2. Integrate relapse prevention strategies into long-term planning.
*Net-Klient Care Management Information System*	Computer Scientist (management information system developer)	Digital case management for ASI-based assessment, follow-up, documentation, decision-support, statistics reporting and inter-organizational collaboration.	1. Conduct and document ASI assessments using Net-Klient. 2. Use digital decision-support for care planning and follow-up. 3. Apply structured digital workflows to support collaboration and systematic monitoring
*Outcome Monitoring and Follow-Up Tools*	Trainers: ASI implementation specialist and management information system developer	Structured outcome monitoring, use of follow-up data for care planning and quality improvement.	1. Apply UBÅT for systematic documentation and outcome monitoring. 2. Use outcome data to inform and adjust care planning.
*Applied Follow-Up and Integrated Care Planning*	Professors and trainers (EBP, implementation, digital practice)	Group-based integration of ASI, MI, relapse prevention, Net-Klient, risk indicators, and UBÅT.	1. Integrate assessment, follow-up, and digital tools into coherent care plans. 2. Apply evidence-based, data-informed reasoning in complex practice scenarios.

### 2.5 Questionnaire development and measures

Prior to the course, participants completed a baseline survey capturing demographic data (e.g., age, gender), professional role, years of experience, and self-reported knowledge regarding harmful substance use and addiction. An initial draft was peer-reviewed by the research team and advisors, then pilot-tested by ten practicing social workers who provided feedback on clarity and user experience. Final revisions were conducted after the on-line feasibility and acceptability tests ensuring that the survey was user-friendly, comprehensive, and appropriately tailored to the intervention group.

### 2.6 Data collection

Data collection included background information, self-assessed competence measures and knowledge assessments, all administered through Survey & Report, Umeå University’s digital survey platform. At baseline (course introduction), participants completed a self-assessed competence questionnaire. Using a 10-point scale (1 = no competence, 10 = fully competent), participants rated their perceived competence across two domains:

1. *working with persons with SUD across ten client group areas*, including those with alcohol use disorders, drug use disorders, mental health problems, children and families, adolescents, co-occurring disorders, older adults, disabilities, criminal justice involvement, and prevention needs (10 items), plus one general item assessing perceived competence in working with vulnerable population groups.
2. *evidence-based screening, assessment, and treatment methods*, including ASI, Documentation system in SUD treatment services (DOK), Alcohol Use Disorders Identification Test (AUDIT), Drug Use Disorders Identification Test (DUDIT), Substance Use Disorder Diagnostic Schedule (SUDDS), cognitive behavioral therapy (CBT), motivational interviewing (MI), relapse prevention (RP), community reinforcement approach (CRA), and individual placement and support (IPS) (10 items), plus one general item assessing perceived competence in evidence-based practices.

### 2.7 Knowledge assessment and scoring

Immediately following the self-assessment of competences, and still prior to course start, participants completed the pre-tests for all course modules. These module-specific pre-tests assessed participants’ initial knowledge related to each module’s content. Throughout the multi-module training program, participants subsequently completed post-tests following the completion of each module. Identical question sets were used for the pre- and post-tests, enabling within-person comparisons of module-specific knowledge acquisition.

The knowledge assessments included both single-response multiple-choice (SRMC) and “select all that apply” multiple-response (SATA MR) multiple-choice items reflecting the theoretical and practical content of the respective module. Each correct response was awarded 10 points, while incorrect responses were scored as 0. When participants skipped individual items, these were also scored as 0; however, module scores were set to missing when all items within a module were unanswered. For multiple-response items, each option was scored independently using a multiple true/false scoring approach (21, 22). Selecting a correct option (key) and correctly not selecting an incorrect option (distractor) each earned 10 points, whereas failing to select a key or selecting a distractor earned 0 points. Total knowledge scores were calculated for each module by summing item scores.

This scoring system was used exclusively for research evaluation and had no impact on participants’ academic standing or course progression. In line with Swedish higher education practices, no numeric grades were reported at course completion. Course completion was determined by submission of a final self-reflective written assignment, which was qualitatively assessed by instructors. Participants (N = 145) whose written assignments met satisfactory standards were deemed to have successfully completed the course.

Several items addressing the historical background of addiction treatment and specific evidence-based practices were closely aligned with pre–post questions developed for a Social Work faculty educational training program conducted in 2017–2018 (13).

### 2.8 Statistical analyses

Demographic and professional characteristics of EPI participants were summarized using descriptive statistics. Data analysis of self-assessed competence was conducted separately from knowledge outcomes, as these measures capture conceptually distinct constructs. The perceived competence analyses focused on two substance-use practice domains described above: competence working with different client groups and evidence-based practices.

Self-assessed competence ratings were treated as ordinal outcomes. Given the skewed univariate distributions of the self-assessed competence ratings, with floor effects for several evidence-based practice items and ceiling effects for client-group items, responses on the 10-point scale were recoded into three categories: Low (1–5), Medium (6–7), and High (8–10). The cut-points were chosen to reflect substantively meaningful positions on the scale (below, just above, and clearly above the midpoint) and were specified prior to fitting the regression models. Ordinal logistic regression models included age, gender, years of experience, and educational background as covariates. Cohort membership was included as a covariate in regression analyses to account for potential temporal or contextual differences across training rounds. Results are presented as adjusted odds ratios (ORs) with 95% confidence intervals (CIs), indicating the likelihood of reporting higher levels of self-assessed competence.

The proportional odds assumption underlying the ordinal logistic regression models was assessed using the Brant test (23). No statistically significant violations of this assumption were detected for most competence outcomes, supporting the use of proportional odds models. However, for competence related to SUDDS, CRA, and IPS, the proportional odds assumption could not be evaluated due to sparse data and low cell counts across several independent variables. These outcomes were therefore excluded from the regression analyses.

Finally, knowledge gains were examined using within-person pre–post comparisons for each module. Changes in knowledge scores were assessed using paired-samples t-tests. In addition to statistical significance testing, the magnitude of change was assessed using Cohen’s d, representing standardized mean change (24, 25). Effect sizes were interpreted using conventional thresholds (d = 0.2 small, d = 0.5 moderate, d = 0.8 large, d = 1.2 very large). For this repeated-measures design, Cohen’s d was calculated as the mean difference divided by the standard deviation of the difference scores, following recommended procedures for within-subject comparisons (26).

## 3 Results

### 3.1 Participant characteristics

**Table 2** summarizes the baseline demographic and professional characteristics of the EPI participants (N = 173) across five admission cohorts. Participants had a mean age of 38.8 years (SD = 9.5), and the majority were women (87.3%), reflecting the gender composition of the Swedish social work profession. Most participants held a university degree (95.4%), and 78.0% had a formal degree in social work.

**Table 2:** Baseline characteristics of EPI training participants (N = 173) by cohort (admission round).

Participant Characteristics	Training cohort						
	AUG 2021	SEPT 2021	JAN 2022	SEPT 2022	JAN 2023	TOTAL	P-values
N	30	20	50	40	33	173	
Gender							
* Man*	6 (20.0%)	1 (5.0%)	7 (14.0%)	2 (5.0%)	6 (18.2%)	22 (12.7%)	0.230
* Woman*	24 (80.0%)	19 (95.0%)	43 (86.0%)	38 (95.0%)	27 (81.8%)	151 (87.3%)	
Age (years)	40.5 (9.6)	40.6 (10.0)	38.5 (8.6)	38.4 (10.8)	37.2 (9.0)	38.8 (9.5)	0.536
Age group							
* 20-29*	1 (3.3%)	3 (15.0%)	9 (18.0%)	8 (20.0%)	5 (15.2%)	26 (15.0%)	0.286
* 30-39*	14 (46.7%)	7 (35.0%)	20 (40.0%)	16 (40.0%)	20 (60.6%)	77 (44.5%)	
* 40-49*	10 (33.3%)	7 (35.0%)	16 (32.0%)	8 (20.0%)	3 (9.1%)	44 (25.4%)	
* 50+*	5 (16.7%)	3 (15.0%)	5 (10.0%)	8 (20.0%)	5 (15.2%)	26 (15.0%)	
Highest education level							
* Upper secondary*	1 (3.3%)	1 (5.0%)	1 (2.0%)	0 (0.0%)	0 (0.0%)	3 (1.7%)	0.237
* Post-secondary (non- university)*	3 (10.0%)	0 (0.0%)	1 (2.0%)	0 (0.0%)	1 (3.0%)	5 (2.9%)	
* University degree*	26 (86.7%)	19 (95.0%)	48 (96.0%)	40 (100.0%)	32 (97.0%)	165 (95.4%)	
Social work degree							
* Yes*	17 (56.7%)	17 (85.0%)	39 (78.0%)	33 (82.5%)	29 (87.9%)	135 (78.0%)	0.028
* No*	13 (43.3%)	3 (15.0%)	11 (22.0%)	7 (17.5%)	4 (12.1%)	38 (22.0%)	
Experience as social worker (years)	9.3 (7.3)	10.6 (7.2)	10.2 (6.3)	9.6 (7.7)	8.4 (8.0)	9.6 (7.2)	0.273
Experience level							
* 0–5 years (early career)*	10 (33.3%)	5 (25.0%)	11 (22.0%)	15 (37.5%)	17 (51.5%)	58 (33.5%)	0.233
* 6–10 years (experienced)*	12 (40.0%)	8 (40.0%)	23 (46.0%)	11 (27.5%)	8 (24.2%)	62 (35.8%)	
* 11+ years (senior)*	8 (26.7%)	7 (35.0%)	16 (32.0%)	14 (35.0%)	8 (24.2%)	53 (30.6%)	
Job title							
* Social worker*	26 (92.9%)	18 (94.7%)	38 (90.5%)	38 (95.0%)	32 (100.0%)	152 (94.4%)	0.131
* Therapist*	2 (7.1%)	1 (5.3%)	4 (9.5%)	0 (0.0%)	0 (0.0%)	7 (4.3%)	
* Other*	0 (0.0%)	0 (0.0%)	0 (0.0%)	2 (5.0%)	0 (0.0%)	2 (1.2%)	

Values are n (%) for categorical variables and mean (SD) for continuous variables.

P-values from Kruskal-Wallis test (continuous) and Pearson chi-squared test (categorical) across training cohorts.

Participants reported an average of 9.6 years of professional experience as social workers. Aside from the proportion with a social work degree, no statistically significant differences were observed across cohorts.

### 3.2 Baseline self-assessed competence across client groups and evidence-based practices

**Figure 1:**
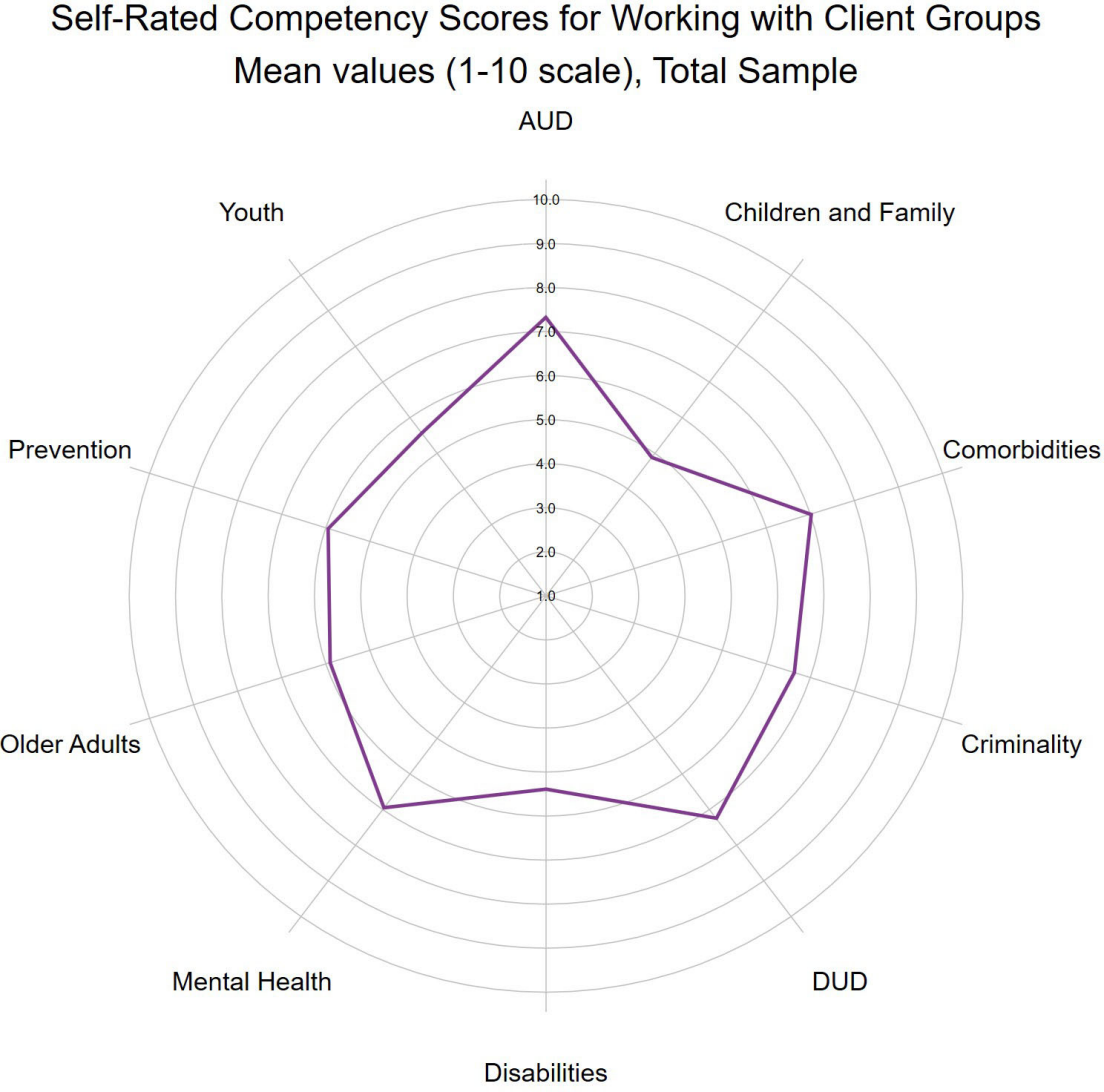
Social workers’ mean self-rated competency for working with substance use disorders across ten practice areas. AUD, alcohol use disorder; DUD, drug use disorder.

**Figure 1** shows baseline self-assessed competence across client groups. Competence was highest for work with alcohol use disorders, drug use disorders, co-occurring disorders, and mental health problems, while lower ratings were reported for children and families, youth, and persons with disabilities. Participants rated their *general* competence in working with vulnerable groups higher than most specific client groups.

**Figure 2:**
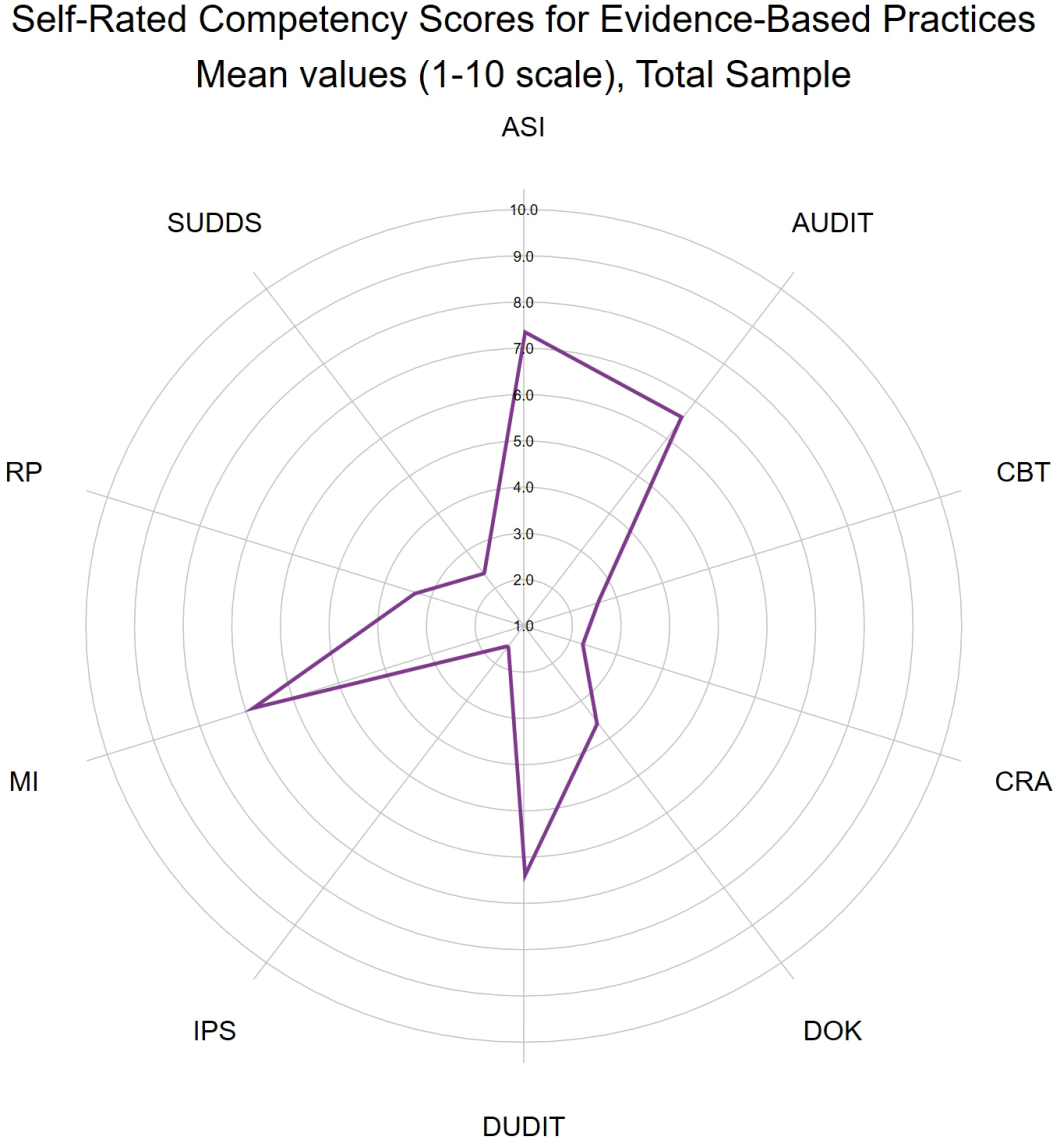
Social workers’ mean self-rated competency across evidence-based screening, assessment tools, and treatment methods. ASI, Addiction Severity Index; AUDIT, Alcohol Use Disorders Identification Test; CBT, Cognitive Behavioral Therapy; CRA, Community Reinforcement Approach; DOK, Documentation system in SUD treatment services; DUDIT, Drug Use Disorders Identification Test; IPS, Individual Placement and Support; MI, Motivational Interviewing; RP, Relapse Prevention; SUDDS, Substance Use Disorder Diagnostic Schedule.

**Figure 2** presents baseline competence in evidence-based practices. Highest ratings were reported for ASI and motivational interviewing, whereas competence in structured psychosocial interventions such as cognitive behavioral therapy (CBT), relapse prevention (RP), community reinforcement approach (CRA), and individual placement and support (IPS) was low.

Ordinal logistic regression analyses (**Tables 3**, **4**) showed that years of professional experience was the most consistent predictor of higher self-rated competence across both client groups and evidence-based practices. Age group and gender were not significantly associated with competence across client groups. For evidence-based practices, participants aged 50 years or older reported lower competence in AUDIT, while no other age or gender effects reached statistical significance. Participants without a formal social work degree reported significantly lower competence in working with older adults, ASI and in evidence-based practices overall. Cohort differences were limited and inconsistent.

**Table 3:** Associations between participant characteristics and self-rated competency for working with SUD across ten practice areas: adjusted odds ratios and 95% confidence intervals from multivariate ordinal logistic regression models.

Variable	AUD	DUD	Mental	Child	Youth	Comorbid	Older	Disability	Crime	Prevention	Vulnerable
Age group (ref: 20–29)											
30–39	1.08 (0.45–2.64)	1.35 (0.56–3.28)	1.17 (0.48–2.88)	1.34 (0.50–3.55)	0.92 (0.38–2.23)	1.30 (0.53–3.18)	1.45 (0.59–3.59)	1.04 (0.42–2.59)	0.95 (0.40–2.24)	1.16 (0.48–2.82)	1.23 (0.49–3.12)
40–49	0.68 (0.23–2.02)	0.86 (0.29–2.56)	0.61 (0.21–1.78)	1.01 (0.31–3.35)	0.37 (0.12–1.11)	0.60 (0.20–1.78)	1.03 (0.35–3.01)	0.47 (0.15–1.47)	0.54 (0.19–1.52)	0.72 (0.24–2.11)	0.85 (0.28–2.61)
50+	0.66 (0.17–2.64)	0.77 (0.19–3.06)	0.38 (0.10–1.46)	1.03 (0.22–4.74)	0.42 (0.10–1.76)	0.78 (0.21–2.88)	2.72 (0.72–10.21)	1.47 (0.36–5.97)	0.71 (0.18–2.73)	1.07 (0.29–3.95)	2.15 (0.48–9.65)
Gender (ref: Man)											
Woman	0.59 (0.22–1.57)	0.63 (0.23–1.67)	0.47 (0.18–1.21)	0.81 (0.30–2.20)	0.74 (0.29–1.87)	0.87 (0.34–2.22)	0.76 (0.30–1.91)	1.06 (0.41–2.70)	1.87 (0.77–4.59)	2.19 (0.85–5.68)	1.58 (0.59–4.25)
Social work degree (ref: Yes)											
No	0.62 (0.28–1.38)	0.65 (0.29–1.43)	1.05 (0.49–2.23)	0.86 (0.35–2.08)	0.67 (0.30–1.49)	1.25 (0.58–2.71)	0.34 (0.16–0.73)**	0.52 (0.23–1.18)	0.93 (0.45–1.92)	1.90 (0.92–3.93)	0.44 (0.19–1.03)
**Experience as social worker in years**	1.10 (1.04–1.17)**	1.10 (1.04–1.17)**	1.07 (1.01–1.13)*	1.02 (0.96–1.08)	1.08 (1.02–1.15)**	1.07 (1.02–1.13)*	1.01 (0.96–1.06)	1.01 (0.95–1.07)	1.06 (1.00–1.11)*	1.02 (0.97–1.08)	1.06 (1.00–1.13)
Cohort (ref: AUG 2021)											
SEPT 2021	0.79 (0.24–2.55)	0.58 (0.18–1.85)	1.38 (0.44–4.30)	1.16 (0.29–4.62)	1.27 (0.39–4.11)	1.46 (0.45–4.70)	1.29 (0.42–4.00)	1.15 (0.35–3.83)	1.22 (0.41–3.64)	0.54 (0.18–1.58)	0.59 (0.18–1.96)
JAN 2022	0.69 (0.27–1.77)	0.73 (0.29–1.86)	0.76 (0.31–1.88)	4.29 (1.45–12.69)**	2.70 (1.07–6.81)*	0.91 (0.36–2.25)	2.92 (1.19–7.20)*	2.51 (0.97–6.53)	1.13 (0.47–2.75)	0.64 (0.26–1.53)	0.94 (0.35–2.57)
SEPT 2022	0.59 (0.22–1.58)	0.64 (0.24–1.69)	0.76 (0.30–1.95)	1.41 (0.43–4.56)	0.86 (0.32–2.31)	0.78 (0.30–2.02)	1.57 (0.60–4.13)	1.62 (0.59–4.48)	0.83 (0.32–2.15)	0.40 (0.16–1.01)	0.41 (0.14–1.15)
JAN 2023	0.51 (0.19–1.43)	0.82 (0.30–2.27)	0.70 (0.26–1.88)	1.88 (0.57–6.16)	1.14 (0.42–3.15)	0.65 (0.24–1.76)	0.77 (0.29–2.07)	1.94 (0.70–5.42)	0.83 (0.32–2.20)	0.39 (0.14–1.06)	1.05 (0.34–3.23)

Self-rated Competency outcomes were measured on a 1–10 scale and recoded into three ordinal categories: Low (1-5), Medium (6-7), and High (8-10) for ordinal logistic regression analyses. AUD, Alcohol Use Disorder; DUD, Drug Use Disorder; Mental, Mental Health; Child, Children and Families; Youth, Adolescents; Comorbid, Co-occurring Disorders/Comorbidities; Older, Older Adults; Vulnerable, Vulnerable population groups in general. Bold values indicate p-value < 0.05.

*p<0.05, **p<0.01.

**Table 4:** Associations between participant characteristics and self-rated competence in evidence-based screening, assessment tools, and treatment methods: adjusted odds ratios and 95% confidence intervals from multivariate ordinal logistic regression models.

Variable	ASI	DOK	AUDIT	DUDIT	CBT	MI	RP	EBP
Age group (ref: 20–29)								
30–39	1.41 (0.55–3.62)	1.56 (0.56–4.30)	1.03 (0.40–2.65)	1.21 (0.47–3.09)	0.55 (0.12–2.54)	1.33 (0.54–3.27)	1.41 (0.35–5.66)	1.74 (0.71–4.26)
40–49	0.57 (0.19–1.74)	0.70 (0.19–2.52)	0.58 (0.19–1.70)	0.69 (0.24–2.03)	0.21 (0.03–1.34)	0.50 (0.17–1.48)	0.91 (0.19–4.36)	1.61 (0.56–4.64)
50+	0.27 (0.07–1.11)	2.01 (0.44–9.18)	0.22 (0.05–0.89)*	0.27 (0.07–1.10)	0.37 (0.04–3.62)	0.63 (0.16–2.50)	1.43 (0.24–8.54)	2.41 (0.58–9.96)
Gender (ref: Man)								
Woman	1.26 (0.50–3.15)	2.75 (0.82–9.21)	0.91 (0.37–2.25)	1.11 (0.45–2.76)	1.04 (0.23–4.68)	0.94 (0.37–2.42)	0.69 (0.22–2.14)	0.66 (0.25–1.76)
Social work degree (ref: Yes)								
No	0.29 (0.13–0.64)**	1.54 (0.65–3.64)	0.62 (0.30–1.28)	0.63 (0.31–1.31)	2.77 (0.95–8.09)	0.81 (0.37–1.76)	2.02 (0.85–4.84)	0.45 (0.21–0.96)*
**Experience as social worker in years**	1.09 (1.03–1.16)**	0.99 (0.93–1.05)	1.07 (1.01–1.12)*	1.06 (1.01–1.12)*	1.12 (1.03–1.22)**	1.10 (1.04–1.17)**	1.08 (1.01–1.15)*	1.04 (0.99–1.10)
Cohort (ref: AUG 2021)								
SEPT 2021	1.80 (0.55–5.86)	0.92 (0.22–3.86)	1.67 (0.51–5.48)	1.66 (0.51–5.43)	0.30 (0.03–3.35)	0.74 (0.23–2.36)	0.55 (0.15–2.04)	0.69 (0.23–2.13)
JAN 2022	0.96 (0.38–2.42)	2.04 (0.71–5.90)	0.97 (0.40–2.35)	0.90 (0.37–2.19)	1.76 (0.44–7.07)	1.19 (0.47–2.98)	0.61 (0.22–1.68)	1.14 (0.47–2.76)
SEPT 2022	2.82 (1.00–7.95)*	1.37 (0.45–4.24)	0.87 (0.33–2.28)	0.88 (0.34–2.29)	0.54 (0.10–2.97)	0.73 (0.28–1.91)	0.26 (0.07–0.90)*	0.61 (0.25–1.54)
JAN 2023	1.51 (0.54–4.25)	1.90 (0.60–5.95)	0.55 (0.20–1.47)	0.57 (0.21–1.52)	0.57 (0.10–3.41)	0.47 (0.17–1.26)	0.31 (0.09–1.09)	0.71 (0.27–1.88)

Self-rated competence outcomes were measured on a 1–10 scale and recoded into three ordinal categories: Low (1–5), Medium (6–7), and High (8–10) for ordinal logistic regression analyses. ASI, Addiction Severity Index; DOK, Documentation system in SUD treatment services; AUDIT, Alcohol Use Disorders Identification Test; DUDIT, Drug Use Disorders Identification Test; CBT, Cognitive Behavioural Therapy; MI, Motivational Interviewing; RP, Relapse Prevention; EBP, Evidence-Based Practices in general. Bold values indicate p-value < 0.05.

*p<0.05, **p<0.01.

### 3.3 Knowledge gains across training modules

**Table 5** summarizes pre–post knowledge scores across assessed modules. Knowledge increased significantly across all modules, with effect sizes ranging from small to very large (d = 0.26–1.41). Lowest baseline scores were observed for *Evidence-Based Biopsychosocial Interventions: Part I* and *Net-Klient Care Management Information System*, which also showed the largest gains. Modules focusing on ASI and professional role in assessment and care planning showed large gains, while smaller effects were observed for the modules *Alcohol and Drug Use: Causes and Consequences* and *Evidence-Based Biopsychosocial Interventions: Part II*. Standard deviations decreased across several modules, indicating greater convergence in knowledge after training.

**Table 5:** Changes in knowledge scores by module.

Module	N.	Question format	Score range	Pre-test mean (SD)	Post-test mean (SD)	Mean difference	Paired t-test	P-value	Cohen’s d
*Professional Role in Assessment and Care Planning*	167	4 SRMC (4 items), 2 SATA MR (8 items)	0 to 120	71.80 (20.60)	87.78 (15.07)	15.99	10.15	<0.001	0.79
*Alcohol and Drug Use: Causes and Consequences*	170	2 SRMC (2 items), 1 SATA MR (6 items)	0 to 80	45.47 (21.95)	51.00 (17.80)	5.53	3.34	0.001	0.26
*Evidence-Based Biopsychosocial Interventions: Part I*	165	2 SRMC (2 items), 1 MRMC (6 items)	0 to 80	28.79 (13.56)	46.42 (12.34)	17.64	15.38	<0.001	1.20
*Evidence-Based Biopsychosocial Interventions: Part II*	161	1 SRMC (1 item), 1 SATA MR (5 items)	0 to 60	41.24 (14.26)	46.21 (9.81)	4.97	4.87	<0.001	0.38
*ASI from a Research Perspective*	166	2 SRMC (2 item), 1 SATA MR (6 items)	0 to 80	58.49 (12.09)	70.72 (10.82)	12.23	11.52	<0.001	0.89
*ASI for Follow-Up Assessment, feedback and clinical decision support*	170	3 SRMC (3 item)	0 to 30	23.88 (8.65)	28.65 (3.91)	4.76	7.24	<0.001	0.56
*Integrating MI and Relapse Prevention in Care Planning*	160	2 SRMC (2 item)	0 to 20	13.75 (7.25)	18.25 (4.13)	4.50	7.95	<0.001	0.63
*Net-Klient Care Management Information System*	163	3 SRMC (3 item)	0 to 30	13.68 (10.18)	28.10 (4.52)	14.42	17.96	<0.001	1.41

Each item scored +10 for correct and 0 for incorrect responses. Cohen’s *d* was calculated for paired samples as the mean difference divided by the standard deviation of the difference scores. Modules focusing on applied, integrative, and reflective learning activities (Modules 9–10) were not assessed using pre–post knowledge tests and are therefore not included.

SATA MR, Select All That Apply Multiple Response Multiple Choice Questions; SRMC, Single Response Multiple Choice Question.

## 4 Discussion

This study examined baseline self-assessed competence in substance use practice among municipal social workers and evaluated knowledge gains following participation in the *Effective Planning of Interventions* (EPI) education, an immersive online educational intervention. Substantial competence and knowledge gaps were observed at baseline, particularly in evidence-based psychosocial interventions and the use of digital systems for client assessment and follow-up. Participation in the online training was associated with meaningful and consistent improvements in knowledge across core domains of substance use practice.

### 4.1 Baseline competence gaps in substance use practice

At baseline, participants reported generally low to moderate self-assessed competence across many client groups and evidence-based practices, despite having, on average, nearly a decade of professional experience. Perceived competence was highest for work with alcohol and drug use disorders, co-occurring disorders, and mental health problems. In contrast, notably lower competence was reported for work with children and families, youth, and persons with disabilities, groups in which substance use is often associated with heightened vulnerability, cumulative disadvantage, and complex service needs. Strengthening social workers’ competencies in recognizing and addressing substance use within these population groups may facilitate earlier identification of substance use problems, improve coordination across service sectors, and support more timely and integrated care responses.

Self-assessed competence was also substantially higher for assessment tools and conversational methods commonly embedded in social work practice, such as the ASI and motivational interviewing, than for structured psychosocial interventions including CBT, relapse prevention, CRA, and IPS. The relatively high baseline competence in ASI likely reflects its long-standing and sustained implementation in Swedish social services since the 1990s, and suggests a successful institutionalization of this assessment practice over time. By contrast, the lower competence reported for structured, manualized psychosocial interventions may reflect the longstanding emphasis within Swedish social work core curricula on assessment, engagement, and case management rather than formal training in specific manualized treatment modalities, alongside limited exposure to specialized post-qualification training within municipal substance use services.

Years of professional experience was the most consistent predictor of higher self-assessed competence both with respect to professional knowledge about different client groups and evidence-based practices. Professional identity in social work is widely understood to be developed over time through practice-based learning, reflection, and professional socialization (27, 28). Although professional identity formation cannot be assessed in this study, the findings underscore the importance of continuity and retention within the municipal social service workforce. This is particularly relevant for the development and maintenance of practice competence in substance use services, where client needs are complex, service systems are fragmented, and workforce turnover is high. This should not, however, be interpreted as evidence that longer professional experience alone ensures evidence-based practice. Experience can generate valuable tacit knowledge, contextual judgement, and professional intuition, but these forms of knowledge need to be combined with formal knowledge, reflection, and analytical scrutiny (29). In the Swedish context, the implementation of ASI was partly intended to reduce unwarranted variation in social work associated with individually based “gut feeling” and to support more systematic, transparent, and comparable assessment practices (30). Assessment and decision-making in social work nevertheless involve practical, moral, and interpretive judgements alongside structured assessment and formal evidence, and practitioners need to integrate experiential knowledge with explicit reasoning and documentation (31).

### 4.2 Knowledge gains following the EPI training

At baseline, pre-EPI participation knowledge assessments revealed substantial gaps in several domains, especially in evidence-based psychosocial interventions and use of digital care management systems. Following participation in the EPI course, statistically significant improvements were observed across all assessed knowledge modules, with effect sizes ranging from small to very large. The magnitude of knowledge gains varied by module content. Modules focusing on digital tools (e.g., the Net-Klient care management information system), evidence-based biopsychosocial interventions, and the professional role of social workers in assessment and care planning were associated with the largest effect sizes. These modules also showed marked reductions in score variability at post-test, suggesting not only increased knowledge but also greater convergence in participants’ understanding following training.

The particularly large gains observed for the Net-Klient module likely reflect low baseline knowledge of digital decision-support systems, despite their widespread availability in Swedish municipal services. This finding is especially relevant in light of the recent Social Services Act reform (SSA 2025:400), which places increased emphasis on systematic follow-up, documentation, and coordination across services. As municipalities are expected to strengthen data-informed practice and inter-organizational collaboration, social workers’ ability to use digital care management systems effectively becomes increasingly central to fulfilling statutory responsibilities. At present, however, digital systems such as Net-Klient primarily support assessment, documentation, and follow-up within municipal social services, while direct electronic sharing of client-level information with regional healthcare providers remains limited by legislative and organizational boundaries. This is especially relevant for individuals with co-occurring psychiatric and substance use conditions who often require parallel interventions from both social and healthcare services. Recent reforms in Sweden promoting more coherent documentation and electronic information exchange between care providers suggest a gradual movement toward improved interoperability. In this context, strengthening social workers’ competence in structured assessment, documentation, and longitudinal data use remains important both for current municipal practice and for future participation in more integrated care pathways.

Similarly, the strong effects observed for modules addressing evidence-based psychosocial interventions mirror the pattern observed in self-assessed competence at baseline, where lower perceived competence was reported for structured, manualized interventions. This gap is particularly salient given the new Social Service Act’s reinforced focus on early, coordinated, and proportionate interventions, which requires social workers to be able to select and align interventions based on assessed needs and follow-up data. Strengthening knowledge of evidence-based psychosocial methods may therefore improve social workers’ capacity to deliver interventions that are effective, appropriate to client needs, and responsive over time within increasingly integrated service systems.

The large knowledge gains observed in the module addressing ASI from a research perspective underscore the importance of strengthening social workers’ understanding of how routine assessment data can be used beyond intake, as a resource for learning, evaluation, and service development. In a learning social service system, systematic documentation and follow-up are not ends in themselves but constitute core mechanisms through which social services are expected to learn from practice, assess whether interventions achieve intended outcomes, and refine service provision over time. Increased competence in interpreting and using longitudinal assessment data may therefore support social workers’ ability to make more evidence-informed decisions and contribute to continuous quality improvement, aligning everyday practice with the Act’s emphasis on effectiveness, prevention, and the use of best available knowledge.

In contrast, modules with a more general or conceptual focus, such as the module on *causes and consequences of alcohol and drug use*, were associated with smaller, but still statistically significant knowledge gains. This finding is consistent with prior research indicating that applied, practice-oriented training tends to produce larger immediate knowledge gains among practitioners than more theoretical content, particularly in continuing professional education contexts (9, 13, 19).

### 4.3 Implications for policy, practice, and education

First, the EPI findings are directly relevant to the new Swedish Social Services Act (SoL 2025:400), which explicitly requires social services to be knowledge-based and of good quality and that municipalities should engage in systematic and continuous follow-up and quality development. Within this policy context, EPI can be understood as a practical capacity-building response that helps municipalities operationalize these statutory requirements through (a) strengthened knowledge of evidence-based assessment and intervention planning, and (b) improved competence in using structured digital systems to support documentation, follow-up, and learning. EPI is also well aligned with the Act’s reinforced emphasis on planning and collaboration, including formal collaboration expectations between municipalities and regions for people with harmful use or substance use disorders. Strengthening staff competence in structured assessment (such as ASI), follow-up logic, and digital competence supports more coordinated care pathways and more coherent information-sharing across organizational boundaries: conditions that are increasingly central to effective collaboration under the new legislative framework (32).

Second, the findings indicate that applied training can address key knowledge gaps and reduce variability in knowledge across practitioners, which is particularly important for maintaining service quality in settings characterized by high caseloads, organizational complexity, and staff turnover. In practice, EPI can be positioned as an organizational learning mechanism that strengthens municipalities’ capacity to (1) use assessment data beyond intake, (2) monitor outcomes more systematically, and (3) align identified needs with appropriate intervention planning. Professional training alone is rarely sufficient for sustained practice change. Implementation research shows that new knowledge is more likely to influence routine practice when training is reinforced by organizational support, quality improvement structures, and continued opportunities to apply and monitor new competencies (33, 34). Practitioners who have acquired new knowledge and skills can only contribute meaningfully to organizational learning and quality improvement when their work is supported by managerial backing, protected time for reflection, access to usable data, feedback loops, and opportunities for collective learning (35–37). Supportive leadership and an organizational culture that values learning are therefore central to sustaining data-informed practice over time.

Third, EPI’s intensive, and fully online format has clear implications for continuing professional development within social services, particularly in systems dominated by generalist social work education. While generalist training provides broad professional foundations, it often offers limited depth in structured, manualized psychosocial interventions and in the applied use of digital decision-support systems in substance use practice. The substantial knowledge gains observed for modules focusing on Net-Klient and evidence-based psychosocial interventions underscore the value of targeted, practice-proximal continuing education that is closely aligned with everyday workflows.

This model of training is especially relevant for:

- rural and geographically dispersed municipalities, where access to in-person education and specialist training is limited.
- services with high caseloads and workforce turnover, where scalable online formats can support more rapid and consistent competence development.
- practitioners with uneven prior training opportunities, where modular designs allow organizations to prioritize core competencies and build knowledge stepwise.

### 4.4 Strengths and limitations

This study has several strengths that support the robustness and relevance of its findings. First, the intervention was implemented at a national scale across 64 municipalities of varying size, organizational structure, and rural–urban distribution, and across five cohorts, increasing ecological validity and relevance for real-world municipal social services. Participants were frontline social workers with statutory responsibility for substance use assessment and service planning, ensuring strong alignment between training content, professional roles, and everyday practice. Second, the EPI modules were developed by an interdisciplinary research team in close collaboration with practitioners, municipal research and development (R&D) representatives, and the Swedish National Board of Health and Welfare, strengthening the practical relevance and policy alignment of the training. Third, the intervention targeted core structural components of substance use practice and combined self-assessed competence measures with objective, module-specific knowledge tests, allowing for a nuanced examination of baseline competence gaps and learning outcomes.

Several limitations should be considered when interpreting the findings. First, the study employed a non-randomized, single-group pre–post design, which limits causal inference, even though the consistent and substantial knowledge gains across modules suggest intervention effectiveness. Second, knowledge gains were assessed using module-specific tests developed for the educational intervention and directly aligned with course content and learning objectives. However, because these were not externally validated instruments, comparability beyond the present context may be limited. In addition, knowledge gains were not assessed through direct observation of practice or behavioral measures. Long-term retention of knowledge and its integration into routine practice were not examined in this article, and the study cannot determine whether knowledge gains translated into sustained changes in assessment quality, intervention planning, or client outcomes. These issues are addressed in subsequent phases of the ESTR research program. Third, participation required managerial approval and access to a specific care management system (Net-Klient), which may limit generalizability to municipalities with different organizational or digital infrastructures and may reflect a more motivated subset of social workers. Finally, although cohort effects were examined, unmeasured contextual factors such as concurrent local initiatives, organizational reforms, or policy changes may have influenced baseline competence or learning outcomes. Future research should also examine implementation fidelity, including the extent to which structured assessments such as ASI continue to be used as intended in routine practice following training.

### 4.5 Conclusions

The findings from the present study suggest substantial variation in municipal social workers’ self-assessed competence across client groups and evidence-based practices, alongside clear gaps in knowledge related to psychosocial interventions and digital systems for assessment and follow-up. Participation in an intensive, online educational intervention was associated with consistent and substantial improvements in knowledge across all assessed domains of the education intervention, with the largest gains observed in education modules where pre-course knowledge levels were lowest. These findings highlight the potential of applied, practice-oriented training to strengthen professional knowledge in areas that are central to contemporary substance use services but insufficiently covered in core social work education and continuing professional development. While further research is needed to examine whether these knowledge gains translate into sustained changes in practice and client outcomes, the results suggest that scalable educational models such as EPI can play an important role in addressing documented knowledge gaps and supporting quality development in municipal social services operating under conditions of high caseloads, workforce turnover, and evolving policy demands.

## Data Availability

The datasets presented in this article are not readily available because The dataset presented in this study is not publicly available because of legal and ethical restrictions. Codes for data preparation and analyses, and output log files can be shared up on request. Requests to access the datasets should be directed to Lena Lundgren, lena.lundgren@umu.se; Wossenseged Birhane Jemberie, wossenseged.jemberie@umu.se.
